# Cancer

**Published:** 1889-10-26

**Authors:** 


### CANCER.

Mr. Alfred Haviland, lecturer on the geographical distribution of disease at St. Thomas's Hospital, remarks on the infrequency of cancer among females in the English lake districts.* Of the inland districts Kendal has the lowest cancer rate. The locality is exposed to the full influence of the prevailing winds from Morecambe Bay, which thoroughly flush the lower valley of the Ken, and thus keep its local climate free from stagnation. If cancer is caused, as recently theorised, by a microbe which gains entrance from without, perhaps this "flushing" may afford some explanation. But we scarcely think it a satisfactory explanation, because the author states that the same district has the highest rate of phthisis. Now phthisis has been attributed to a microbe, and if flushing took one microbe away, there is no reason why it should not dispose of another species of microbe. The author some time back attempted to demonstrate that cancer among females has a well-defined geographical distribution throughout England and Wales. Roughly speaking, the conclusions were that the highest mortality from cancer occurs in low-lying districts liable to be flooded, while the lowest mortality occurred on high average levels, and where the rock base below the soil consists of red sandstone, mountain limestone, or chalk. There are, however, very few maladies which are not more prevalent and more destructive to life in damp, malarious, low-lying localities than on dry, open, airy sites. It is found that the annual death rate from cancer to every 10,000 women living in England and Wales is 14·31, and in the lake district 11·50, which the author calculates would equal a saving of 170 lives during 20 years. We do not, however, place very much confidence in statistics of the kind, principally because registration is not sufficiently advanced to afford a firm basis. It cannot be doubted that various maladies are returned as cancer which really are not so. There are few maladies more difficult to diagnose than cancer of the breast, for instance, at least in the earlier stages, and when the malady is of an internal organ the difficulties are increased. It has been recently stated that cancer is on the increase in this country, but this apparent increase may be due to more accurate diagnosis.

